# Pulsed Light treatment below a Critical Fluence (3.82 J/cm^2^) minimizes photo-degradation and browning of a model Phenolic (Gallic Acid) Solution

**DOI:** 10.3390/foods8090380

**Published:** 2019-09-01

**Authors:** Artur Wiktor, Ronit Mandal, Anika Singh, Anubhav Pratap Singh

**Affiliations:** 1 Food, Nutrition and Health, University of British Columbia, 2205, East Mall, Vancouver, BC V6T 1Z4, Canada; 2 Faculty of Food Sciences, Department of Food Engineering and Process Management, Warsaw University of Life Sciences (WULS-SGGW), Nowoursynowska 159c, 02-776 Warsaw, Poland

**Keywords:** pulsed light, fluence, gallic acid, non-thermal treatment

## Abstract

Pulsed light (PL) is one of the most promising non-thermal technologies used in food preservation and processing. Its application results in reduction of microbial load as well as influences the quality of food. The data about the impact of PL on bioactive compounds is ambiguous, therefore the aim of this study was to analyze the effect of PL treatment of a gallic acid aqueous solution—as a model system of phenolic abundant liquid food matrices. The effect of PL treatment was evaluated based on colour, phenolic content concentration and antioxidant activity measured by DPPH assay using a design of experiments approach. The PL fluence (which is the cumulative energy input) was varied by varying the pulse frequency and time. Using Response Surface Methodology, prediction models were developed for the effect of fluence on gallic acid properties. It was demonstrated that PL can modify the optical properties of gallic acid and cause reactions and degradation of gallic acid. However, application of PL did not significantly alter the overall quality of the model gallic acid solution at low fluence levels. Cluster analysis revealed that below 3.82 J/cm^2^, changes in gallic acid were minimal, and this fluence level could be used as the critical level for food process design aiming to minimize nutrient loss.

## 1. Introduction

For a long time, the food industry has been using thermal methods like pasteurization, sterilization, etc. for preservation of foods and extension of their shelf life. However, thermal processing operations have drawbacks associated with them. For instance, due to the high processing temperature, the nutrients may be destroyed. Also, the sensory characteristics may be affected [1]. Modern day consumers are more aware than ever before. They continuously demand food which is safe, of good eating quality and nutritionally sound. This has led the food processing scientists to seek and research food processing methods which can make the food safe, while keeping the nutritional properties intact.

Pulsed light (PL) technology has now been widely explored as a novel non-thermal food preservation method that uses a form of energy other than heat for achieving food preservation. PL uses high-intensity short duration white light (wavelength of 200–1100 nm) for microbial inactivation [2]. The electrical energy is stored in capacitors and discharged in short bursts or pulses of high intensity. The ultra-violet (UV) fraction of the spectrum is associated with microbial inactivation as well as other chemical changes in food products.

A multitude of phenolic compounds like ellagic acid, ferulic acid, gallic acid, etc. are synthesized by plants’ fruits, vegetables, as part of their secondary metabolism. In the early 1960s these phenolic compounds were considered as by-products of the plant metabolism, which were present in the vacuoles of cells. These compounds act as complex constituents of pigments, antioxidants, flavoring agents, in plants and plant-based foods. Thus, they form a major part of our diet. Apart from that, they are also bioactive compounds that are anti-inflammatory, anticarcinogenic, can decrease blood sugar levels, reduce body weight and ageing [3].

The light sensitivity of phenolics is a topic of utmost importance for studying the processing of foods using light. The photoinduced degradation of gallic acid (a model system representing plant phenolics) was reported earlier in [4,5]. Benitez et al. [5] demonstrated that gallic acid subjected to UV radiation degrades following first-order kinetics reaction. Thus, UV radiation could be used in the wastewater treatment process after cork production. However, the authors pointed out that UV- provoked photolysis of gallic acid was a rather slow process—after 90 min of radiation the concentration of gallic acid decreased from 50 to 10–40 ppm, depending on pH. Also, the progress of the process may be different depending on the wavelength spectrum of the light used. A photoinduced decrease in total phenolic content was also observed for real food systems, i.e., pineapple juice subjected to UV-C treatment with a dose 10.76 mJ/cm^2^ [6] or pumelo juice treated with a UV-C dose of 15.45–27.63 mJ/cm^2^ [7]. It should be emphasized therefore that the data about the impact of UV light treatment of food on its bioactive compounds is ambiguous. There are articles which report no significant changes of phenolics after exposure of juices or solid food matrices to UV light or PL [8,9,10]. Similar statements can be made regarding the antioxidant activity. Thus, the data concerning the impact of PL on model systems becomes even more important to understand the basic mechanisms of effects and in designing proper PL processing systems for foods rich in phenolics.

PL technology is still in its infancy and therefore there are a limited number of studies that have been carried out on the effect of PL on nutrient attributes, while most available studies focus on the microbiological safety aspect of PL [11]. It is a matter of immense importance that this novel process ensures food safety while retaining the bioactive compounds in food and keeping the sensory properties intact. To the best of our knowledge, there is no literature available on the effect of PL on model polyphenolic solutions like gallic acid. Also, the literature on the effect of PL on liquid foods is scarce. In accordance, the aim of this study was to evaluate the effect of PL processing on the physicochemical properties of a model gallic acid solution using an experimental setup designed for thin-profile treatment of liquid foods.

## 2. Materials and Methods

### 2.1. Material

Gallic Acid (3,4,5-trihydroxybenzoic acid; Sigma Aldrich Co., Oakville, ON, Canada) was used to prepare a model solution with a concentration of 0.5 mg/mL. Methanol (≥99.8%) and Folin-Ciocalteu reagents were purchased from Merck KGaA (Darmstadt, Germany). Ethanol, 2,2-diphenyl-1-picrylhydrazyl (DPPH) free radical and Na_2_CO_3_ were purchased from Alfa Aesar, Thermo Fisher Scientific Chemicals, Inc. (Ward Hill, MA, USA). (±)-6-Hydroxy-2,5,7,8- tetramethylchromane-2-carboxylic acid (Trolox^TM^) was purchased from Sigma-Aldrich Co. (Oakville, ON, Canada).

### 2.2. Pulsed Light Equipment

**Figure 1:**
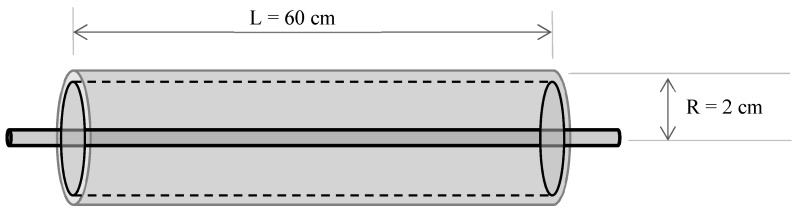
PL processing chamber. The chamber has an annular design inside which the liquid is housed and treated by PL lamps placed at the axis of chamber. Suitable for batch or continuous processing.

The experiments were carried out in a bench-top pulsed light equipment designed at the Faculty of Land and Food Systems, University of British Columbia in collaboration with Solaris Disinfection Inc. (Mississauga, ON, Canada). The equipment consisted of two parts: (1) a cylindrical annular chamber built of quartz glass for thin profile liquid treatment. The chamber has an inlet and an outlet for flowing liquid in and out of the chamber; (2) a xenon flashlamp placed at the axial center of the cylindrical chamber that emits light pulses for liquid treatment. The annular volume of the treatment chamber was 75 mL and its average distance from the lamp axis was 2 cm. The liquid thickness in the chamber was around 1 mm. Pulsed light lamps emitted 30 J of light energy per pulse (comprising wavelengths ranging from far UV to near IR in the electromagnetic spectrum) on a chamber area (impact surface) of 675 cm^2^. A schematic diagram of the equipment is given in Figure 1.

### 2.3. Design of Experiment

Table 1 shows the plan of the experiments. The response surface methodology approach was used for the experimental design to evaluate the effect of pulsed light treatment parameters on the physicochemical properties of gallic acid solutions. The central composite rotatable design (CCRD) used (α = k^1/2^ with two numeric factors k = 2 − frequency of pulses and treatment time) was prepared utilizing Statistica 13 Design (Statsoft Inc., Tulsa, OK, USA). It was composed of 10 experimental trials with two separate replicates in the central point (run 9 (C) and 10 (C)). Frequency varied from 1 to 10 Hz whereas time ranged from 5 to 50 s.

Energy input (in terms of fluence) depended on the parameters pulse frequency and treatment time and it was equal to 1.07–17.2 J/cm^2^, since the device applied 30 J with one single pulse regardless of the frequency. The boundary conditions of treatment were selected based on literature data considering the energy necessary to inactivate microorganisms [11,12]. Each run was performed in two separate replicates, which means that central point was repeated four times in total. The responses described in subsequent sections were evaluated for these treatments and also for the untreated sample.

### 2.4. Temperature Increment Measurement

The emitted light energy was absorbed by the solution as heat. The amount of heat absorbed was calculated for each run. The temperature changes for the gallic acid solution were recorded immediately after each treatment. A temperature measuring RTD (ThermoProbe Inc., Jackson, MS, USA) was used to measure the temperature. The initial solution temperature was recorded to be 21.2 °C. The measurements were duplicated for each observation and the temperature change % and heat gain [13] were calculated using Equations (1) and (2):

Temp. increase % = (T_f_ − T_i_)/T_i_ × 100 (1)

Heat gain (J/cm^2^) = [4.19 × (T_f_ − T_i_) × V × ρ]/A (2)

where, T_f_, T_i_ are final and initial temperature (°C); V = chamber volume (m^3^); ρ = liquid density (kg/m^3^), A = area [cm^2^]. The constant 4.19 (kJ/kg-K) is taken as the specific heat of the gallic acid solution which is assumed equal to that of water [13].

### 2.5. Colour

The colour of the treated and untreated samples was measured using a colorimeter (HunterLab, model LabScan^TM^ XE Plus, Hunter Associates Laboratory, Reston, VA, USA). Colour was expressed in CIE L* (whiteness or brightness), a* (redness/greenness) and b* (yellowness/blueness) coordinates. Two replicate measurements were performed, and results were averaged. The total colour difference (Δ*E*) and browning index (BI) were calculated [14] using the following Equations (3) and (4):

ΔE = ((L − L_o_)^2^ + (a − a_o_)^2^ + (b − b_o_)^2^)^1/2^ (3)

BI = 100 × (x − 0.31)/0.172(4)

where:

x = (a* + 1.75 × L*)/(5.645 × L* + a* − 3.012 × b*)(5)

In Equation (3), the L_o_, a_o_ and b_o_ are the colour values for untreated samples, and the constants in Equations (4) and (5) were taken from the literature [14].

### 2.6. Total Phenolic Content, Gallic Acid Content and Antioxidant Activity Determination

Total phenolic content (TPC) were estimated using the Folin–Ciocalteau’s (FC) method with modifications [15]. Briefly, an aliquot (5 mL) of the gallic acid solution was transferred to a glass tube; reactive 10^−1^ diluted FC reagent (20 mL) is added after 5 min; sodium carbonate (Na_2_CO_3_, 5 mL, 7.5% *w/v*) was added and the mixture shaken. After 30 min of incubation at ambient temperature in the dark, 200 µL samples were placed in 96-well plates. Finally, the absorbances were measured in a spectrophotometer (Infinite Pro M200 series, Tecan^TM^, Männedorf, Switzerland) at 765 nm and compared to a gallic acid calibration curve for TPC (prepared using 0 to 1 mg/mL concentration gallic acid solution). Results were expressed as mg gallic acid equivalent (GAE)/100 mL. All measurements were done in duplicate.

Gallic acid content (GAC) was determined using HPLC (Agilent 1100 system, Agilent Technologies, Santa Clara, CA, USA) equipped with a Zorbax SB-C18 column according to the methodology presented in [16]. This was carried out to measure the changes in gallic acid concentration due to photodegradation. Results were expressed as mg GAC/100 mL solution.

To determine the antioxidant activity (AA) of gallic acid solutions, 2,2-diphenyl-1-picrylhydrazyl (DPPH) free radical scavenging assay was used. A standard curve was constructed using Trolox^TM^ (20 µM) solution. For sample wells, gallic acid (20 µL) was added. In both standard and sample wells of a 96-well microtiter plate, 1 mM DPPH (20 µL) was added. The blank well consisted of HPLC grade methanol (200 µL). The plate was incubated for 10 min at room temperature in the dark. Then the plate absorbances were read at 519 nm by a microtiter plate reader (Tecan^TM^ Infinite M200 Pro). All reagents were dissolved in HPLC grade methanol. Antioxidant capacity reported in mM Trolox^TM^ equivalents (TE) per mL of solution.

### 2.7. Statistical Analysis

All the data were expressed as mean ± SD after carrying out technical and biological replicate experiments. Tukey’s test was used to test for differences at a significance level of *p* ≤ 0.05 where appropriate. The Pearson’s correlation analysis was employed to assess the relationship between selected parameters and variables. The comprehensive statistical analyses of all obtained results were performed by Hierarchical Cluster Analysis using Ward method. The significance of the impact of pulsed light treatment parameters was evaluated using response surface methodology (RSM) approach. All statistical analyses were performed using Statsoft Inc’s Statistica 13 software (Tulsa, OK, Canada). 

## 3. Results

### 3.1. Impact of PL on the Temperature Increment of Gallic Acid Solutions

**Figure 2:**
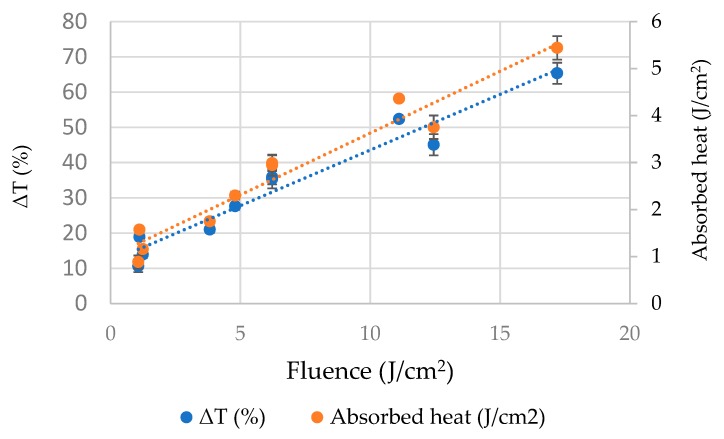
The impact of PL on temperature increment of gallic acid aqueous solution.

This test was carried out to quantify the energy imparted to gallic acid solutions by PL application. The heat energy absorbed by gallic acid solution and thereby temperature increment due to volumetric heating showed a proportionality with the fluence delivered. There was a strong and significant positive correlation between the temperature increment % and fluence of PL treatment as shown in Figure 2 (r = 0.974; *p* < 0.05). The temperature increment was the lowest (10.6%) in case of fluence level of 1.07 J/cm^2^. Similarly, the increment was highest (65.3%) in case of highest fluence level of 17.2 J/cm^2^. The heat absorbed by gallic acid solution after PL application varied from 0.884 to 5.44 J/cm^2^.

The temperature increment during PL treatment can be attributed to energy absorption by samples by virtue of the photothermal effect, which shows an increase in temperature by light absorption. Whenever light interacts with a sample, it decays exponentially as per the Beer-Lamberts law and thus get converted into heat energy in the sample [2]. Similar observations have been made by researchers doing experiments with milk [17], fruit juices [18].

### 3.2. Colour Measurement of PL Treated Gallic Acid Solution

**Figure 3:**
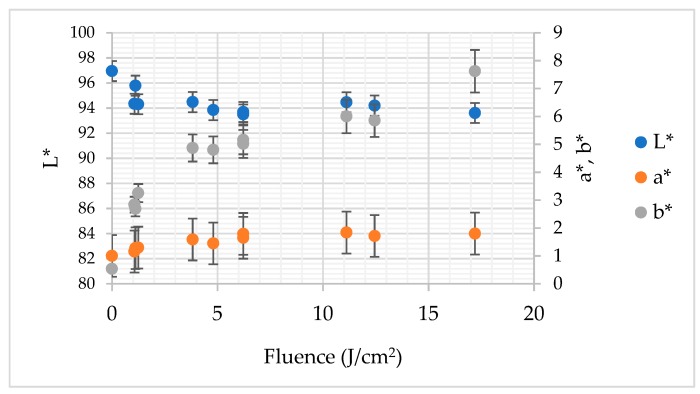
The impact of pulsed light on L*, a* and b* colour parameters of gallic acid aqueous solution.

The L* and b* colour parameters of all PL treated gallic acid solutions were significantly different (*p* < 0.05) from that of untreated sample (Figure 3). In the case of the a* coordinate the vast majority of the PL-treated samples exhibited significantly different values. Only the sample treated by 1.070 J/cm^2^ did not differ from the control. More specifically, the L* parameter was equal to 93.49–95.78 and 96.96 in the case of PL treated and untreated solution, respectively. In the case of the a* colour parameter, which represents the share of red and green colour, the changes were smaller. For instance, the untreated gallic acid solution was characterized by a* = 1000 whereas PL application resulted in increment of this coordinate to 1.84 and 1.80 in the case of fluence 11.10 and 17.20 J/cm^2^, respectively. The difference between these samples expressed by changes of red/green share was statistically irrelevant (*p* > 0.05). As aforementioned, the b* component of colour of gallic acid solution was significantly affected by PL application. The highest fluence (17.20 J/cm^2^) resulted in the biggest change of blue/yellow colour share −7.62. For comparison, the b* of untreated gallic acid solution was equal to 0.54. 

**Figure 4:**
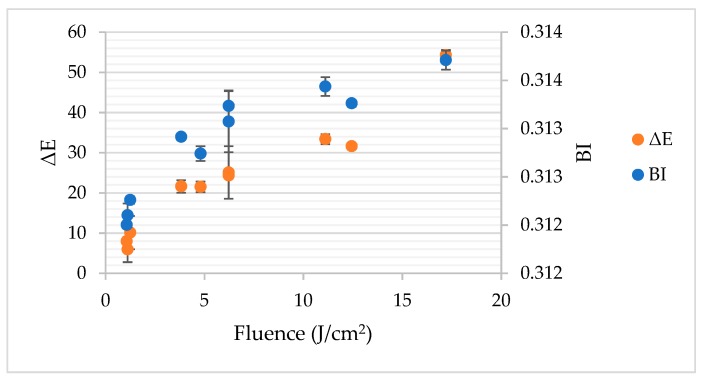
The impact of pulsed light on total colour difference (ΔE) and browning index (BI) of gallic acid aqueous solution.

The Pearson’s correlation analysis proved that the relation between a* and fluence had significant and positive character with *r* = 0.820 (*p* < 0.05). In turn, the Pearson’s correlation coefficient established for the relation between b* and fluence was even bigger—*r* = 0.950 (*p* < 0.05). To the contrary, no significant linear dependency was found between fluence and L* (*r* = −0.456; *p* > 0.05). The changes of optical parameters clearly demonstrate that PL provoked browning of gallic acid solution. The total colour difference (ΔE) and browning index (BI) depended strongly on fluence as presented in Figure 4. The Pearson’s correlation coefficient for the relationship with fluence was equal to *r* = 0.967 and *r* = 0.902, for ΔE and BI, respectively. These changes of colour may be attributed to the photodegradation of gallic acid by the UV light component of PL [19]. The possible mechanism that could be involved in browning may be related to the production of reactive oxygen species (ROS) due to the presence of oxygen in the gallic acid solutions. ROS may oxidize gallic acid and form quinones or semiquinones [20]. These products may undergo further different reactions, i.e., polymerization, and form brown pigments [21]. The temperature increment may also play a role in browning of gallic acid solution, since the correlation between colour changes expressed by BI and ΔE was significant (*p* < 0.05) and positive (*r* = 0.926 and *r* = 0.953, respectively).

### 3.3. Impact of PL on Total Polyphenol Content and Antioxidant Activity Expressed by DPPH Assay

Table 2 presents the gallic acid content (GAC), total phenolics content (TPC) and antioxidant activity of all investigated gallic acid solution samples. Almost all samples treated by PL exhibited smaller concentrations of gallic acid as measured by the HPLC method. However, the difference was significant only for samples that were treated by 3.82 J/cm^2^. In this case the concentration of gallic acid was equal to 43.15 mg/100 mL, which means that it was 13.7% lower in comparison to untreated solution. Interestingly, the delivery of higher fluence did not necessarily cause higher degradation of gallic acid as measured by the HPLC method against TPC. It could indicate that there exist some optimal parameters for gallic acid decomposition by light. 

These results are very interesting especially when compared to TPC results since they indicate that even if the gallic acid content was not changed, the TPC was altered. The TPC of control sample was 50.6 mg GAE/100 mL. All samples subjected to PL treatment were characterized by significantly (*p* < 0.05) smaller TPC which ranged between 41.27 and 47.23 mg GAE/100 mL. The biggest decline of TPC, equal to 18.4% in comparison to the reference sample, was found for the trial with the highest delivered fluence (17.2 J/cm^2^).

The smallest applied fluence (1.07 J/cm^2^) also contributed to a significant decrease of TPC by 6.6% when compared to the control solution. It means that the reactions gallic acid can undergo are very photosensitive. Even though there was no degradation of gallic acid under this condition some compounds that could react with FC reagent were formed. However, within the fluence range of 1.07–12.44 J/cm^2^ the TPC remained the same from statistical point of view. It means that despite high photosensitivity of gallic acid, there is a wide spectrum of fluence that cause similar degradation level. Such information is extremely important when designing PL-based technology of processing phenolic-abundant products. It is worth emphasizing that the degradation of gallic acid maintained a negative linear relation with fluence. The Pearson’s correlation coefficient for this dependency was *r* = −0.856 (*p* < 0.05). What’s more, the relationship between TPC and b*, ΔE and BI was significant as well (*p* < 0.05) and correlation coefficients were equal to −0.771; −0.851 and −0.654, respectively. Indeed, it indicates that products of degradation of gallic acid contribute directly to browning of the investigated solution [4]. The photoinduced decomposition of gallic acid was also reported by Benitez et al. [5].

Further, it must be mentioned that we observed the measured TPC was slightly lower than the measured gallic acid content for all samples. Ideally, as the model gallic acid solution did not had any other component, the TPC should be equal to the gallic acid content. It has been previously reported [22] that radiation by UV, which is also a constituent of pulsed light, may create reactive oxygen species by photooxidation of oxygen which is dissolved in water. These intermediates can form other reactive molecules like hydroxyl radicals and hydrogen peroxide, which included with the products of photodecomposition of gallic acid, have been reported to interfere with the Folin–Ciocalteu reagent. For instance, hydrogen peroxide can lead to lower TPC values if present in the system together with gallic acid, as found by Rangel et al. [23].

In the current study, almost all samples exhibited antioxidant activity similar to control solution. The decrease of free radical scavenging activity was found only for sample treated by the highest fluence – 17.2 J/cm^2^. Comparing these results with gallic acid concentration it can be stated that within the fluence range of 1.07 to 12.4 J/cm^2^ the products of gallic acid oxidation exhibited some antioxidant potential. Similar results were reported by Oms-Oliu et al. [24] for mushrooms subjected to PL. The authors have found that depending on the fluence the antioxidant activity can be decreased or increased which is related with decomposition of phenolics and polymerization reaction of the quinones. Also, Llano et al. [25] reported that application of PL with fluence 8–16 J/cm^2^ helps to maintain the antioxidant activity of fresh-cut apples.

### 3.4. Response Surface Methodology (RSM) and Cluster Analysis

**Figure 5:**
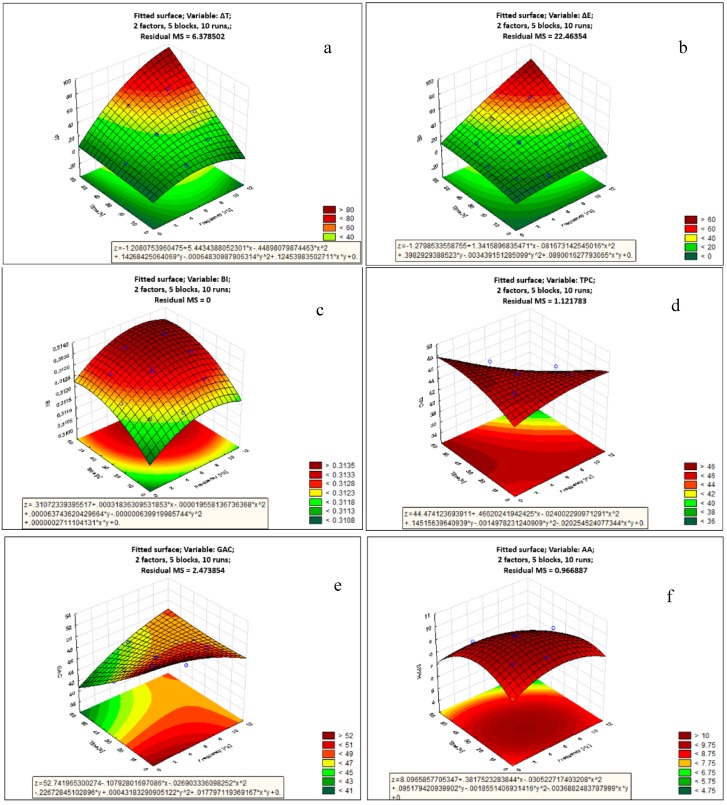
RSM plots showing the impact of PL on the properties of gallic acid aqueous solution. from top to bottom: (**a**) temperature change, ΔT; (**b**) total colour difference, ΔE; (**c**) browning index, BI; (**d**) Total phenolic content, TPC; (**e**) Gallic acid content, GAC; (**f**) antioxidant capacity.

RSM analysis was applied to estimate the values of investigated variables of gallic acid solution depending on frequency (x) and time (y) of PL treatment. Equations (6–11) depict the response model equation, wherein, a significant model was obtained for all responses, with a high coefficient of regression *R*^2^ (>0.9) for temperature increment, color change and browning index responses. The gallic acid content model showed lowest *R*^2^ (0.74), whereas R^2^ of total phenolic content and antioxidant activity were within acceptable range (0.8–0.9). The results of the analysis of variance (ANOVA) for the response surface models are given in Table 3. The model itself was found significant for all responses (*p* < 0.05) and was adequate for navigating the design space. The plots of RSM have been given in Figure 5:

ΔT (%) = −1.208 + 5.443x − 0.449x^2^ + 0.143y − 0.0006y^2^ + 0.1245xy, *R*^2^ = 0.98 (6)

ΔE = −1.279 + 1.342x − 0.082x^2^ + 0.398y − 0.0034y^2^ + 0.089xy, *R*^2^ = 0.95 (7)

BI = 0.311 + 0.0003x − 0.00002x^2^ + 0.0001y − 0.000001y^2^ + 0.0000003xy, *R*^2^ = 0.99 (8)

TPC (mg GAE/100 ml) = 44.474 + 0.466x − 0.240x^2^ + 0.145y − 0.0015y^2^ − 0.0202 xy, *R*^2^ = 0.82 (9)

GAC (mg/100 mL) = 52.742 − 0.108x − 0.027x^2^ − 0.227y − 0.0004y^2^ + 0.018xy, *R*^2^ = 0.74 (10)

AA (mM TEAC/mL) = 8.096 + 0.382x − 0.031x^2^ + 0.095y − 0.0019y^2^ − 0.004xy, *R*^2^ = 0.88 (11)

Based on the ANOVA results in Table 3, it could be concluded that individual interactions of both PL frequency (x) and PL treatment time (y) significantly affected (*p* < 0.005) temperature change, total color change and browning index. Based on the signs of the corresponding coefficient in Equations (6)–(8), it could be concluded that higher PL frequency and higher PL time led to higher values of temperature change, total color change and browning index. Total phenolic content was significantly affected by only PL frequency (0.005 < *p* < 0.05) and not PL treatment time (*p* > 0.05), whereas gallic acid content and antioxidant activity were significantly affected by only PL treatment time (0.005 < *p* < 0.05) and not PL frequency (*p* > 0.05).

The effect of the mutual interaction of PL frequency and PL treatment time (x*y), which describes the PL fluence that governs the microbial lethality level during the PL process, was only significant (0.005 < *p* < 0.05) for temperature change, and did not affect other responses. Based on this, it could be inferred that even a process requiring high fluence could be optimized to minimize quality loss (at same levels of microbial lethality) by understanding the relative importance of PL frequency and time on the response. The effect of the quadratic interaction of PL frequency (x^2^) was only significant (0.005 < *p* < 0.05) for temperature change and browning index, while that of PL treatment time (y^2^) was only significant (0.005 < *p* < 0.05) for antioxidant activity and browning index. This is often indicative of the curvilinear relationship between the parameters.

Based on the total sum of squares in Table 3 for significant interactions, effect of PL treatment time dominated the value of all responses except temperature increment and total phenolic content as compared to PL frequency. Thus, PL treatment time must be minimized preferably over PL treatment time for reducing color change, gallic acid deterioration, browning index and antioxidant activity.

**Figure 6:**
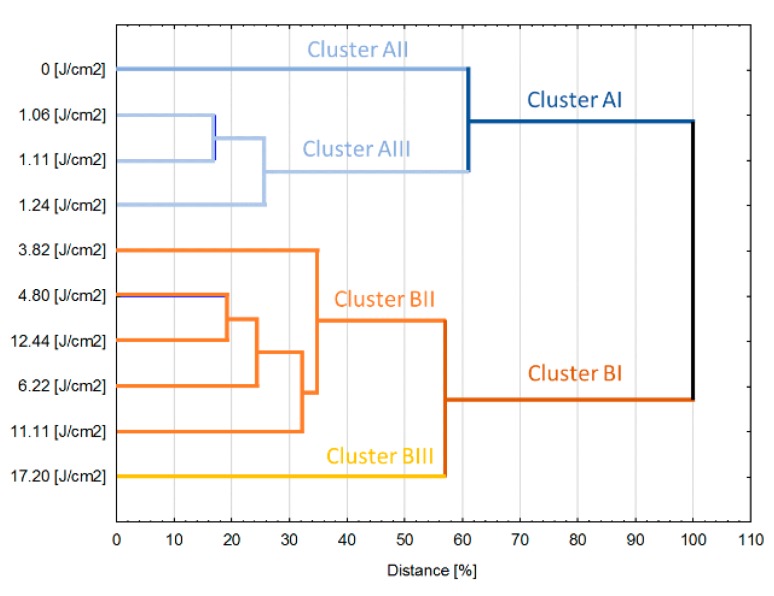
Results of the cluster analysis performed based on of all investigated variables.

Cluster analysis (Figure 6) allowed the samples to be distinguished into two big aggregates depending on the fluence. Cluster AI was formed by the reference gallic acid solution and samples treated by rather small fluences <3.82 J/cm^2^. In turn, all samples that were treated by higher energies were gathered in Cluster BI. Within the clusters, the samples also exhibit some dissimilarity—in Cluster AI, the reference sample formed separate, one item aggregate while in the Cluster BI, similar behavior was observed for sample treated by the highest fluence. Based on that data it can be estimated that when it comes to treatment of polyphenols rich liquid food, there is a threshold value of fluence which does not lead to relevant quality changes. Results obtained for gallic acid model solution indicate that treatment below 3.82 J/cm^2^ maintains the quality (expressed by the investigated physicochemical properties) almost unchanged from the statistical point of view whereas delivery of higher than 3.82 J/cm^2^ values of fluence lead to relevant modification of the quality. A similar optimization approach of imposing limits on reciprocation time and frequency was proposed in our previous work [26] for improving the quality of reciprocating agitation thermal processing, which suggests the parallel between optimization approaches for both thermal and non-thermal processing technologies. What more, the application of fluence higher than 11.1 J/cm^2^ results in severe modification of the quality giving the product with significantly different properties than solutions treated by other parameters. However, it is worth emphasizing that obtained results are valid for model solution and should be rather considered as leads when it comes to the design and analysis of PL treatment of real food systems.

## 4. Conclusions

Pulsed light (PL) processing happens to be a promising non-thermal technology for food preservation and processing. It has been shown to have great potential for decontaminating food products by destroying pathogenic microorganisms. However, the effect of PL on food bioactive and nutritional compounds is unclear. The aim of this study was to understand how the PL treatment affects the gallic acid aqueous solution, which is taken as a model system of phenolic-abundant liquid food matrices. Several parameters of the gallic acid solution were tested by a design of experiments approach for the effect of PL processing-colour, total phenolic concentration, antioxidant activity using DPPH free radical assay. It was found that PL can modify the optical properties of gallic acid and cause reactions and degradation of gallic acid. The absorbed light energy resulted in a proportional temperature increase. The L* and b* color values decreased significantly, with an increase in the browning index on PL treatment. The gallic acid content and the total polyphenol content changed initially, but remained constant after a critical fluence was reached, whereas the antioxidant activity did not vary significantly, except at the highest fluence level tested (17.20 J/cm^2^). This suggests that despite the photosensitivity of the gallic acid, the degradation products still had similar antioxidant potential even at relatively high fluences. Based on a cluster analysis, a critical fluence level (<3.82 J/cm^2^) was suggested, below which PL shall have minimal effect on the overall quality of model gallic acid solution. With less than 10% degradation of gallic acid and less than 20% degradation of the antioxidant activity, it can be said that the PL technology has excellent ability to process liquid foods rich in polyphenols and antioxidants.

## Figures and Tables

**Table 1:** The set-up of the performed experiment with fluence during PL treatment.

Run *	Factor A		Factor B		Fluence (J/cm^2^)
	Coded Value	Frequency (Hz)	Coded Value	Time (s)	
1	−1	2	−1	12	1.1
2	−1	2	1	43	3.8
3	1	9	−1	12	4.8
4	1	9	1	43	17.2
5	−1.41	1	0	28	1.2
6	1.41	10	0	28	12.4
7	0	5	−1.41	5	1.1
8	0	5	1.41	50	11.1
9 (C)	0	5	0	28	6.2
10 (C)	0	5	0	28	6.2

* Where (C) is the center point of the design. Additionally, untreated samples were also evaluated for the same responses.

**Table 2:** Total phenolics content (TPC) and antioxidant activity measured by DPPH assay due to PL treatment.

Run	Fluence (J/cm^2^)	TPC (mg GAE/100 mL) *	GAC (mg /100 mL) *	Antioxidant Activity (mM TEAC/mL) *
Untreated	0.0	50.6 ± 1.07 ^a^	50.00 ± 0.58 ^ab^	10.29 ± 0.88 ^a^
1	1.07	47.23 ± 0.33 ^b^	50.64 ± 2.32 ^b^	9.55 ± 1.01 ^ac^
2	3.82	46.63 ± 0.57 ^b^	43.15 ± 0.78 ^c^	9.4 ± 0.5 ^abc^
3	4.80	45.99 ± 1.19 ^b^	49.67 ± 0.88 ^ab^	9.26 ± 0.01 ^bc^
4	17.20	41.27 ± 0.37 ^c^	46.65 ± 0.74 ^ac^	8.22 ± 0.76 ^b^
5	1.24	46.8 ± 1.32 ^b^	48.02 ± 0.87 ^ab^	9.29 ± 0.17 ^abc^
6	12.44	44.82 ± 0.37 ^b^	47.90 ± 0.85 ^ab^	9.31 ± 0.22 ^abc^
7	1.11	45.77 ± 0.97 ^b^	49.83 ± 0.86 ^ab^	9.75 ± 0.35 ^a^
8	11.11	45.61 ± 1.11 ^b^	47.44 ± 0.86 ^ab^	9.27 ± 0.92 ^bc^
9 (C)	6.22	45.99 ± 0.59 ^b^	48.02 ± 0.85 ^ab^	9.96 ± 0.21 ^a^
10 (C)	6.22	46.47 ± 0.56 ^b^	47.89 ± 0.86 ^ab^	9.92 ± 0.09 ^a^

* Values expressed as mean ± SD followed by letters a–c wherein, means followed by same letter are not significantly different (*p* > 0.05).

**Table 3:** Analysis of Variance (ANOVA) table for the influence of PL frequency and time on the physicochemical properties of gallic acid solution.

**Parameter**	**Temperature Change (% ΔT)**			**Total Color Change (ΔE)**			**Browning Index (BI)**		
	Type III SS	F Value	Pr > F	Type III SS	F Value	Pr > F	Type III SS	F Value	Pr > F
Model	2.82 × 10^3^	-	3.23 × 10^−5^ ***	1.85 × 10^3^	-	1.70 × 10^−3^ **	3.09 × 10^−6^	-	8.70 × 10^−15^ ***
Frequency (x)	1.38 × 10^3^	2.17 × 10^2^	1.24 × 10^−4^ ***	7.48 × 10^2^	3.33 × 10	4.47 × 10^−3^ **	1.10 × 10^−6^	1.51 × 10^2^	2.53 × 10^−4^ ***
x^2^	1.01 × 10^2^	1.59 × 10	1.63 × 10^−2^ *	3.35	1.49 × 10^−1^	7.19 × 10^−1^	1.92 × 10^−7^	2.64 × 10	6.79 × 10^−3^ *
Time (y)	1.22 × 10^3^	1.92 × 10^2^	1.57 × 10^−4^ ***	9.53 × 10^2^	4.24 × 10	2.87 × 10^−3^ **	1.76 × 10^−6^	2.42 × 10^2^	9.96 × 10^−5^ ***
y^2^	1.25 × 10^−1^	1.96 × 10^−2^	8.96 × 10^−1^	3.51	1.56 × 10^−1^	7.13 × 10^−1^	1.22 × 10^−7^	1.67 × 10	1.50 × 10^−2^ *
x*y	1.85 × 10^2^	2.89 × 10	5.77 × 10^−3^ *	9.43 × 10	4.20	1.10 × 10^−1^	8.75 × 10^−10^	1.20 × 10^−1^	7.46 × 10^−1^
**Parameter**	**Total Phenolic Content (TPC)**			**Gallic Acid Content (GAC)**			**Antioxidant Activity (AA)**		
	Type III SS	F Value	Pr > F	Type III SS	F Value	Pr > F	Type III SS	F Value	Pr > F
Model	2.56 × 10	-	4.39 × 10^−7^ ***	3.87 × 10	-	1.80 × 10^−6^ ***	3.31	-	1.51 × 10^−6^ ***
Frequency (x)	1.13 × 10	1.00 × 10	3.39 × 10^−2^ *	6.55 × 10^−1^	2.65 × 10^−1^	6.34 × 10^−1^	2.75 × 10^−1^	2.84	1.67 × 10^−1^
x^2^	2.90 × 10^−1^	2.58 × 10^−1^	6.38 × 10^−1^	3.64 × 10^−1^	1.47 × 10^−1^	7.21 × 10^−1^	4.68 × 10^−1^	4.84	9.26 × 10^−2^
Time (y)	4.62	4.11	1.12 × 10^−1^	2.16 × 10	8.72	4.19 × 10^−2^ *	1.44	1.49 × 10	1.82 × 10^−2^ *
y^2^	6.66 × 10^−1^	5.94 × 10^−1^	4.84 × 10^−1^	5.54 × 10^−2^	2.24 × 10^−2^	8.88 × 10^−1^	1.02	1.06 × 10	3.13 × 10^−2^ *
x*y	4.88	4.35	1.05 × 10^−1^	3.77	1.52	2.85 × 10^−1^	1.62 × 10^−1^	1.67	2.65 × 10^−1^

* *p* < 0.05; ** *p* < 0.005; *** *p* < 0.0005.
